# The orally active and bioavailable ATR kinase inhibitor AZD6738 potentiates the anti-tumor effects of cisplatin to resolve ATM-deficient non-small cell lung cancer *in vivo*

**DOI:** 10.18632/oncotarget.6247

**Published:** 2015-10-27

**Authors:** Frank P. Vendetti, Alan Lau, Sandra Schamus, Thomas P. Conrads, Mark J. O'Connor, Christopher J. Bakkenist

**Affiliations:** ^1^ Department of Radiation Oncology, University of Pittsburgh School of Medicine, Pittsburgh, PA, USA; ^2^ Cancer Bioscience, AstraZeneca, Macclesfield, United Kingdom; ^3^ Women's Health Integrated Research Center at Inova Health System, Department of Defense Gynecologic Cancer Center of Excellence, Annandale, VA, USA; ^4^ Department of Pharmacology and Chemical Biology, University of Pittsburgh School of Medicine, Pittsburgh, PA, USA

**Keywords:** ATM and Rad-3-related (ATR), ataxia telangiectasia mutated (ATM), cisplatin, non-small cell lung cancer (NSCLC), DNA damage response

## Abstract

ATR and ATM are DNA damage signaling kinases that phosphorylate several thousand substrates. ATR kinase activity is increased at damaged replication forks and resected DNA double-strand breaks (DSBs). ATM kinase activity is increased at DSBs. ATM has been widely studied since ataxia telangiectasia individuals who express no ATM protein are the most radiosensitive patients identified. Since ATM is not an essential protein, it is widely believed that ATM kinase inhibitors will be well-tolerated in the clinic. ATR has been widely studied, but advances have been complicated by the finding that ATR is an essential protein and it is widely believed that ATR kinase inhibitors will be toxic in the clinic. We describe AZD6738, an orally active and bioavailable ATR kinase inhibitor. AZD6738 induces cell death and senescence in non-small cell lung cancer (NSCLC) cell lines. AZD6738 potentiates the cytotoxicity of cisplatin and gemcitabine in NSCLC cell lines with intact ATM kinase signaling, and potently synergizes with cisplatin in ATM-deficient NSCLC cells. In contrast to expectations, daily administration of AZD6738 and ATR kinase inhibition for 14 consecutive days is tolerated in mice and enhances the therapeutic efficacy of cisplatin in xenograft models. Remarkably, the combination of cisplatin and AZD6738 resolves ATM-deficient lung cancer xenografts.

## INTRODUCTION

ATR and ATM are apical DNA damage signaling kinases that phosphorylate a broad and overlapping catalogue of several thousand substrates [1–3]. ATR kinase activity is increased at damaged replication forks and resected DNA double-strand breaks (DSBs) [4]. ATM kinase activity is increased at DSBs [5]. ATM has been widely studied since individuals with the disease ataxia telangiectasia, who express no ATM protein, are the most radiosensitive patients identified [6, 7]. Lung cancer cells are radiosensitized by pharmacologic ATM kinase inhibitors in tissue culture [8–10], and it is widely believed that ATM kinase inhibitors will be well-tolerated in the clinic as ATM is not an essential protein [7, 11]. However, ATM kinase inhibition does not phenocopy ATM protein disruption, and while *atm*–/– mice that express no ATM protein are viable, mouse embryos expressing kinase-inactive ATM protein die before embryonic day 9.5–10.5 [12–14]. It is therefore important to investigate the impact of ATM and ATR kinase inhibitors in preclinical models.

ATR has also been widely studied, but advances have been complicated by the finding that ATR is an essential protein in mice and mammalian cells [15–18]. Overexpression of kinase-inactive ATR increases sensitivity to cisplatin and ionizing radiation (IR) in tissue culture [19, 20] and, consistent with these data, lung cancer cells are sensitized to cisplatin and IR by ATR kinase inhibitors *in vitro* [21–26]. ATR kinase activity is increased after hypoxia and ATRi's sensitize radiation-resistant hypoxic cells to IR [25, 27–29]. Furthermore, ATR kinase inhibitors synergize with loss of ERCC1, ATM, XRCC1, and DNA damaging chemotherapy agents in tissue culture [26, 30, 31]. While these *in vitro* data advance ATR kinase inhibitors for the treatment of lung cancer, there is a pervasive view that ATR kinase inhibitors will be toxic in the clinic.

VX-970 (also referred to as VE-822), the first bioavailable ATR kinase inhibitor described, was shown to enhance the therapeutic efficacy of IR and gemcitabine in xenograft models of pancreatic cancer [32]. In these experiments, VX-970 was administered orally daily for 6 consecutive days. VX-970 was also shown to enhance the therapeutic efficacy of cisplatin in patient-derived lung tumor xenografts [33]. In these experiments, VX-970 was administered orally for 4 consecutive days per week. VX-970 is in clinical trials, but is not orally administered to human subjects.

Here we describe AZD6738, an orally active and bioavailable ATR kinase inhibitor that is also in clinical trials and is orally administered. These trials will assess safety of AZD6738 alone and in combination with radiotherapy as well as chemotherapy. We show here that AZD6738 induces cell death and senescence in non-small cell lung cancer (NSCLC) cell lines. Furthermore, AZD6378 potentiates the cytotoxicity of cisplatin and gemcitabine in NSCLC cell lines in which ATM kinase signaling is intact, and potently synergizes with cisplatin to kill ATM-deficient NSCLC cells *in vitro*. We show that daily administration of AZD6738 for 14 consecutive days is tolerated in mice and that AZD6738 enhances the therapeutic efficacy of cisplatin in xenograft models. Remarkably the combination of cisplatin and AZD6738 resolves ATM-deficient lung cancer xenografts.

## RESULTS

### AZD6738 is a highly selective and potent inhibitor of ATR kinase activity

**Figure 1:**
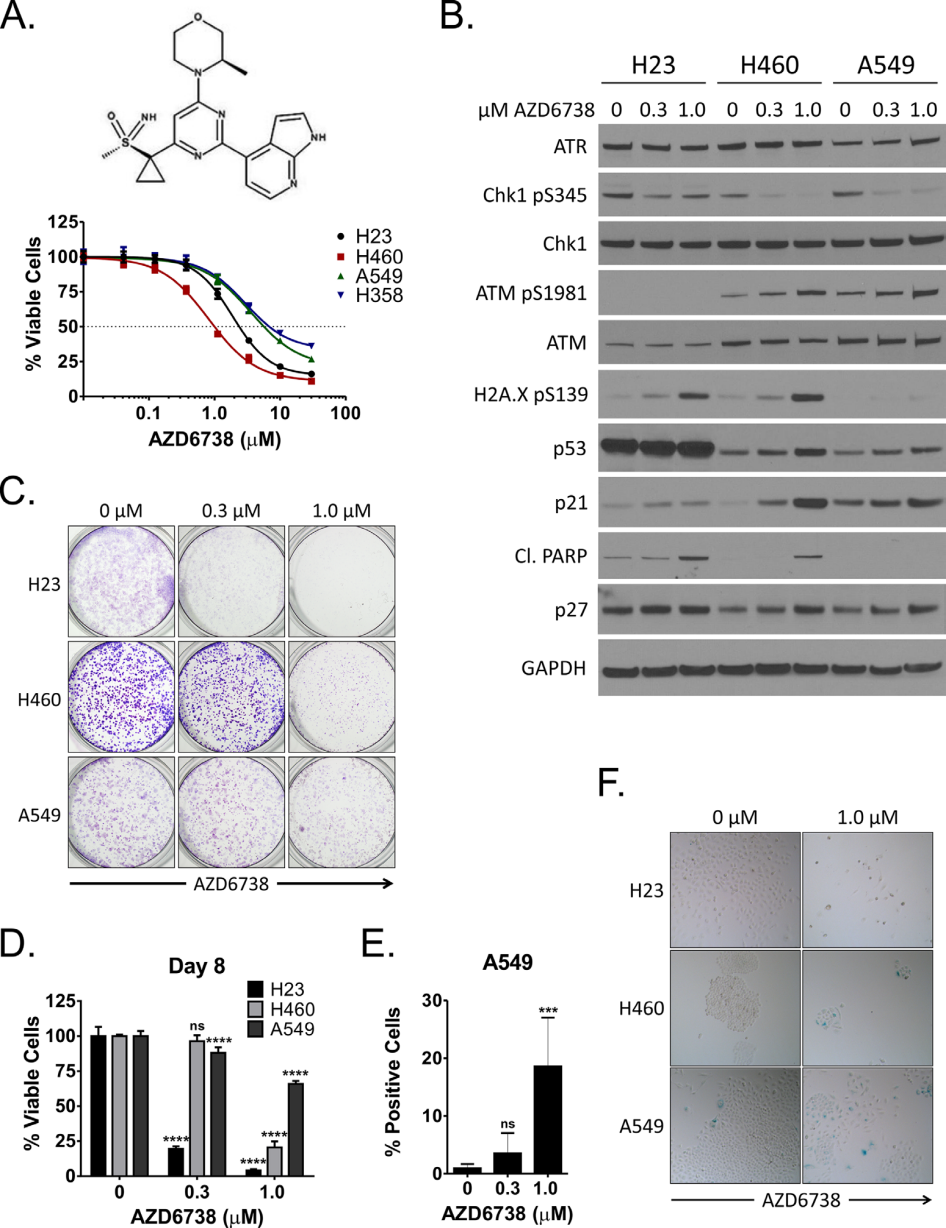
Inhibition of ATR by AZD6738 inhibits growth of NSCLC cells and induces a DNA damage response **A.** Log dose response curves for NSCLC cell lines (H23, H460, A549, H358) treated with AZD6738 for 48 hours. Curves from representative experiments with 5 replicates per dose tested and depict the mean percentage of viable cells (± SD) relative to the mean of control cells. **B.** Western blots for ATR, phospho-Chk1 (S345), total Chk1, phospho-ATM (S1981), total ATM, phospho-H2A.X (S139), p53, p21, cleaved PARP, and p27 following 24 hour treatment of H23, H460, and A549 cells with 0.3 μM or 1.0 μM AZD6738. **C.** H23, H460, and A549 cells were treated for 48 hours with 0.3 μM or 1.0 μM AZD6738 and incubated in drug-free media for an additional 3 (H460, A549) or 4 (H23) days. Cells were then stained with crystal violet to visualize colony formation. **D.** H23, H460, and A549 cells were treated for 48 hours with 0.3 μM or 1.0 μM AZD6738, harvested, and re-seeded at equal density in 96-well plates. Cells were grown an additional 6 days in the absence of AZD6738 and viability was assessed on day 8. Bars represent the mean percentage of viable cells (± SD) relative to the mean of control cells, averaged from 2 independent experiments, each with 4 replicates per condition (*n* = 8 total). Statistical significance by ANOVA with Dunnett's multiple comparison test denoted as follows: ****P ≤ 0.0001, ns (not significant). **E–F.** H23, H460, and A549 cells were treated for 48 hours with 0.3 μM or 1.0 μM AZD6738 and incubated in drug-free media for an additional 2–3 days. Cells were then stained for senescence associated β-galactosidase activity. E. Quantitation of SA-β-gal positive A549 cells at day 5. Bars represent the mean percentage of positive cells/field (± SD), averaged from 2 independent experiments, each with 3 fields/replicate and 3 replicates per condition (*n* = 18 fields total). Statistical significance by ANOVA with Dunnett's multiple comparison test denoted as follows: ***P ≤ 0.001, ns (not significant). F. Representative images (20x objective) of SA-β-gal staining in H23 (day 5), H460 (day 4), and A549 (day 5) following treatment with 1.0 μM AZD6738.

AZD6738 is a potent inhibitor of ATR kinase activity with an IC_50_ of 0.001 μM against the isolated enzyme and 0.074 μM against ATR kinase-dependent CHK1 phosphorylation in cells (structure shown in Figure 1). The identification and characterization of AZD6738 will be described in detail elsewhere. Briefly, AZD6738 was screened against a 71 kinase panel including related PI3K and protein PI3K-like kinases (ATM, DNA-PK and mTOR) in *in vitro* isolated enzyme assays using ^32^P radioactive assays to determine potency and selectivity. A large margin of activity was observed relative to ATR enzyme isolated activity (0.001 μM) for most targets tested with the closest targets being PI3Kδ at 6.8 μM (6800-fold above ATR IC_50_) and DYRK at 10.8 μM (10800-fold) (AstraZeneca, personal communication). Kinase selectivity was also determined using active-site dependent competition binding assays against 442 targets at 1 μM AZD6738 with only PI3KC2G showing any significant inhibition (20%) (Astra Zeneca, personal communication).

AZD6738 was screened for inhibition of closely related PI3K target pathway signaling in cell assays to determine potency and selectivity. A large margin of activity was observed for all targets tested relative to inhibition of ATR kinase-dependent kinase signaling (0.074 μM), with ATM, DNA-PK, and PI3Kα kinase inhibition all > 30 μM, and mTOR kinase inhibition > 23 μM (Supplementary Table S1).

### AZD6738 inhibits ATR kinase activity and impairs viability of NSCLC *in vitro*

We first examined whether AZD6738 has single agent activity against NSCLC cell lines *in vitro*. We assessed ATP levels (using the CellTiter-Glo Luminescent Cell Viability Assay) as a surrogate for cell viability following 48 hour treatment with 0 μM–30 μM AZD6738 in four *Kras* mutant cell lines: H23, H460, A549, and H358. AZD6738 impaired viability of these cells lines, with the lowest GI_50_ and greatest maximal inhibition in H460 and H23 cells (1.05 μM, 88.0% and 2.38 μM, 86.2%, respectively) (Figure 1A, Supplementary Table S2).

We next examined the effects of AZD6738 on DNA damage response signaling in H23, H460, and A549 cells (Figure 1B). Following treatment for 24 hours with 0.3 or 1.0 μM AZD6738, ATR kinase activity was inhibited in all three cell lines, as evident by a decrease in Chk1 phosphorylation (S345) without change in total ATR or Chk1 protein levels. In p53-wildtype H460 and A549 cells, AZD6738 induced activation of ATM (S1981 phosphorylation), stabilization of p53, and expression of p21 and p27, in a dose dependent manner. Increased ATM kinase activity in cells treated with ATR kinase inhibitors has been reported previously [23, 26]. Importantly, H23 cells, which possess a missense mutation in ATM (Q1919P) [34], exhibited minimal total ATM protein and no phospho-ATM (S1981). Expectedly, AZD6738 did not induce p21 expression in H23 cells which also harbor a p53 mutation (M246I) [34]. Similarly, p27 levels remained largely unchanged in response to treatment. However, H23 and H460, but not A549, exhibited a marked increase in phospho-H2A.X (S139) and cleaved PARP, indicative of accumulation of DNA damage and induction of apoptosis, respectively. This coincides with the observed greater sensitivity of H23 and H460 in viability assays.

To better understand whether the effects of AZD6738 treatment on cell viability persist after relief of ATR inhibition, we first treated cells with 0.3 or 1.0 μM AZD6738 for 48 hours. Following treatment, cells were either grown in drug-free media for an additional 3–4 days to form colonies or re-seeded in 96-well plates at equal density for each treatment condition and grown in drug free media for six days prior to assessment of viability on day 8. Colony formation was markedly reduced in all cell lines treated with 1.0 μM AZD6738, particularly H23 and H460, and in H23 treated with the 0.3 μM (Figure 1C). Similarly, despite re-seeding, 0.3 and 1.0 μM AZD6738 treatment resulted in a dramatic decrease in viable cell yield at day 8 in H23 (19.5% and 4.1% of control, respectively, *P* ≤ 0.0001), while 1.0 μM AZD6738 reduced yield of H460 and A549 cells to 24.0% and 65.9% of control, respectively (*P* ≤ 0.0001) (Figure 1D). Treatment with 0.3 μM AZD6738 resulted in a modest reduction in viable cell yield in A549 (88.0% of control, *P* ≤ 0.0001), but not H460.

Recent work has shown that inhibition of ATR kinase, caused by an inactivating mutation in the ATR activation domain (AAD) of TOPBP1, an allosteric activator of ATR kinase activity, caused cellular senescence in mouse embryonic fibroblasts [35]. Several CDK inhibitors are key mediators of cellular senescence, including p16, p21, and p27 [36–38]. In A549 and H460 cells, neither of which express p16 due to homozygous deletion of *CDKN2A* [39, 40], we noted induction of p21 and p27 in response to AZD6738 treatment. In addition, A549 cells did not exhibit increased PARP cleavage, suggesting an alternative mechanism to apoptosis may be responsible for phenotype observed in our long term assays. We assessed senescence associated β-galactosidase (SA-β-gal) activity 2–3 days after treatment with AZD6738 (Figure 1E–1F and Supplementary Figure S1). Consistent with a lack a p21 and p27 induction in H23 cells, we saw no evidence of increased senescence at day 5 with AZD6738 treatment. Conversely, we noted an approximately 19.7-fold increase in SA-β-gal positive A549 cells (day 5) following treatment with 1.0 μM AZD6738. While 0.3 μM AZD6738 increased SA-β-gal activity by approximately 3.7-fold, the difference was not statistically significant. We also observed an increase in SA-β-gal activity in H460 cells (day 4). However, similar to the colony formation experiments, there was a dramatic decrease of overall cell number after treatment with 1.0 μM AZD6738 (compared to control). This dose resulted in ~50% inhibition in our 48 hour dose-response studies, as well as PARP cleavage by 24 hours. Taken together, these data suggest that senescence is a contributing mechanism, but not the predominant mechanism, responsible the impairment of H460 growth and viability.

### AZD6738 strongly synergizes with cisplatin in an ATM-deficient NSCLC cell line *in vitro*

Recent studies have demonstrated combinatorial activity of ATR kinase inhibitors with DNA damaging agents in both *in vitro* and *in vivo* solid tumor models, including NSCLC [33, 41]. To screen for potential synergistic dose combinations of AZD6738 with the standard-of-care chemotherapeutic agents cisplatin, gemcitabine, and docetaxel, we treated H23, H460, A549, and H358 cell lines in a 2-dimensional dosing matrix and determined the excess inhibition over Loewe additivity for each dose combination in the matrix [42]. Loewe excess matrices indicated combinatorial activity of AZD6738 with cisplatin and gemcitabine, but not docetaxel, in all cell lines (Supplementary Figure S2).

**Figure 2:**
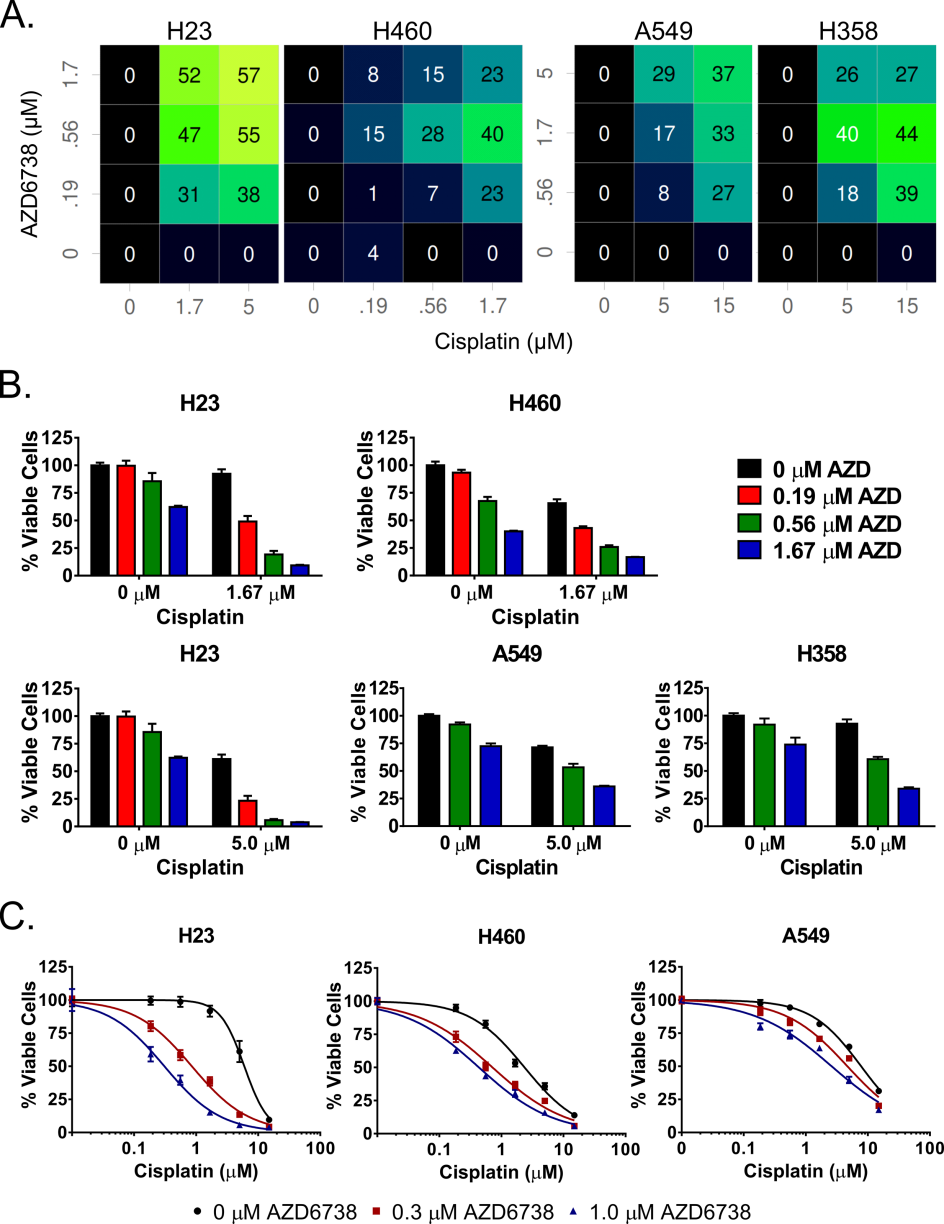
AZD6738 sensitizes NSCLC cell lines to cisplatin and synergizes strongly with cisplatin in ATM-deficient H23 cells **A–B.** Cells were treated with select doses of AZD6738 and cisplatin (as indicated) for 48 hours and viability was assessed at the end of treatment. A. Color coded matrix displays the inhibition in excess of Loewe additivity, with brighter colors and corresponding higher inhibition values indicative of greater synergy. B. Bars represent the mean percentage of viable cells (± SD) relative to the mean of control cells, averaged from 2 independent experiments, each with 3–4 replicates per condition (*n* = 7–8 total). **C.** Shift in cisplatin sensitivity in H23, H460, and A549 cell lines upon addition of 0.3 or 1.0 μM AZD6738. Curves represent the mean percentage of viable cells (± SD) relative to the mean of 0 μM cisplatin controls within each AZD6738 treatment condition. Data averaged from 2 independent experiments, each with 3 replicates per condition (*n* = 6 total).

To validate combinatorial activity of AZD6738 with cisplatin and gemcitabine, we selected dose combinations concentrated in areas of excess inhibition in the Loewe excess matrices. We assessed ATP levels as a surrogate for cell viability by CellTiter-Glo following 48 hour treatment with the selected doses. The selected combinations of AZD6738 and cisplatin resulted in additive to synergistic inhibition of viability in all cell lines (Figure 2A–2B). Striking synergy was observed in the ATM-deficient H23 cells, with doses of 0.19, 0.56, and 1.67 μM AZD6738 and 1.67 and 5.0 μM cisplatin. Synergy was also observed in H358, and to a lesser degree, A549 cells, but at higher doses of both AZD6738 (0.56, 1.67 and 5.0 μM) and cisplatin (5.0 and 15.0 μM). In addition, the overall effects on cell viability were much less than observed in H23 cells (Figure 2B). As H460 cells exhibited higher sensitivity to both agents alone, lower dose ranges for each agent (0.19–1.67 μM) were employed. The selected combinations yielded additivity to moderate synergy. We also observed synergy with select dose combinations of AZD6738 and gemcitabine in all four cell lines (Supplementary Figure S3). However, the degree of synergy and effect on cell viability was less pronounced in H23 compared to the synergy observed with cisplatin.

Next we sought to determine to what degree AZD6738 shifts sensitivity to cisplatin in H23, H460, and A549 cells. We treated cells for 48 hours with a fixed 0.3 or 1.0 μM dose of AZD6738 and a dose range of cisplatin (0.19–15.0 μM) and assessed viability by CellTiter-Glo. We observed dramatic shifts in cisplatin sensitivity in H23 cells compared to H460 and A549 cells (Figure 2C). Treatment with 1.0 μM AZD6738 resulted in a 19.41-fold decrease in cisplatin IC^50^ in H23 cells versus 5.44- and 2.99-fold in H460 and A549 cells, respectively (Supplementary Table S3). Similarly, 0.3 μM AZD6738 caused a 7.00-fold shift in H23, compared to 3.29- and 1.66-fold in H460 and A549, respectively.

### The combination of AZD6738 and cisplatin promotes accumulation of cells at the G1/S border and in early S-phase

**Figure 3:**
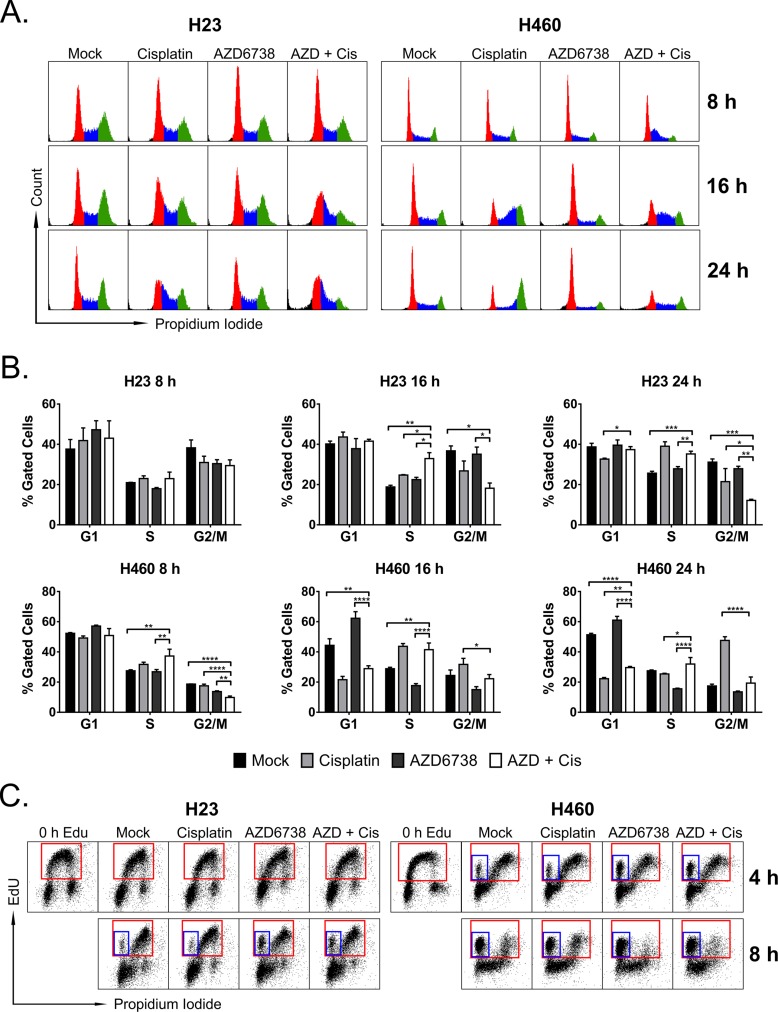
The combination of AZD6738 and cisplatin causes accumulation of cells in early S-phase and at the G1/S border **A–B.** Cells were treated with 1.0 μM AZD6738, 5.0 μM (H23) or 1.67 μM (H460) cisplatin, combination, or mock control for the durations indicated, and cell cycle profiles were determined using propidium iodide staining of DNA content. A. Representative cell cycle profiles for H23 (left) and H460 (right) cells following 8, 16, and 24 hour treatment with AZD6738, cisplatin, combination, or mock. B. Quantitation of the percentage of cells in G1, S, and G2/M phases of the cell cycle at the specified time points. Bars represent the mean percentage of gated cells (± SD). Data averaged from 2 independent experiments, each with 1–2 replicates per condition. Statistical significance by ANOVA with Tukey's multiple comparison test denoted for AZD + Cis compared to other treatments as follows: *P ≤ 0.05, **P ≤ 0.01, ***P ≤ 0.001, ****P ≤ 0.0001. **C.** Cells were pulsed with 10 μM EdU for 15 min (H23) or 10 min (H460) and then treated with AZD6738, cisplatin, combination, or mock for 4 or 8 hours. Representative dot plots show EdU incorporation versus DNA content (stained with propidium iodide), with red boxes denoting cells that have incorporated EdU, and blue boxes denoting early S-phase cells that have incorporated EdU but have not progressed in S-phase (eg. have not increased DNA content).

To determine whether the combination of AZD6738 and cisplatin resulted in cell cycle perturbations in H23 and H460 cells, we assessed cell cycle profiles at 8, 16, and 24 hours after treatment with 1.0 μM AZD6738, 5.0 μM (H23) or 1.67 μM (H460) cisplatin, combination, or mock (Figure 3A). In H23 cells, by 16 hours combination treatment resulted in accumulation of cells at the G1/S border (32.9% vs. 18.8% cells in S-phase, *P* ≤ 0.01, compared to mock), and loss of cells in G2/M (18.2% vs. 36.8%, *P* ≤ 0.05, compared to mock) (Figure 3B). By 24 hours, cells treated with the combination exhibited further reduction in the G2/M population (12.1% vs. 31.1%, *P* ≤ 0.001, compared to mock), persistence at the G1/S border (35.2% vs. 25.7% cells in S-phase, *P* ≤ 0.001, compared to mock), and an increase in the sub-G1 population. H23 cells treated with cisplatin alone now also exhibited accumulation in early S-phase (39.1% vs. 25.7%, *P* ≤ 0.0001, compared to mock).

Conversely, H460 cells treated with the combination of AZD6738 and cisplatin exhibited early S-phase accumulation (37.3% vs. 27.5%, *P* ≤ 0.01) and loss of cells in G2/M (9.8% vs. 18.7%, *P* ≤ 0.0001) by 8 hours, compared to mock control (Figure 3B). Combination treatment resulted in loss of cells in G1 (28.9% vs. 44.3%, *P* ≤ 0.01, compared to mock) and accumulation in S-phase (41.5% vs. 28.8%, *P* ≤ 0.01, compared to mock), in conjunction with the emergence of a sub-G1 population by 16 hours. AZD6738 treatment alone triggered G1 accumulation (62.3% vs. 44.3%, *P* ≤ 0.001, compared to mock), loss of cells from S-phase (17.7% vs. 28.8%, *P* ≤ 0.01, compared to mock), and emergence of a sub-G1 population by 16 hours. Conversely, cells treated with cisplatin alone began accumulating in late S-phase (43.7% vs. 28.8%, *P* ≤ 0.001, compared to mock). While cisplatin treatment resulted in clear G2/M arrest by 24 hours, combining AZD6738 with cisplatin abrogated this G2/M arrest (*P* ≤ 0.0001, compared to cisplatin alone), and resulted in an overall loss of cycling cells and persistence of a sub-G1 population.

To better determine the effects of AZD6738, with and without cisplatin, on S-phase progression, we pulsed H23 and H460 cells with 10 μM EdU prior to treatment as described above for 4 or 8 hours. Compared to mock and cisplatin treatment, AZD6738 and combination treatment caused accumulation of cells at the entry into S-phase in both cells lines, as evident by the population of cells that have incorporated EdU but have not increased DNA content beyond that of G1 cells (Figure 3C). Treatment with AZD6738 and combination resulted in an increase from 3.7% (mock control) to 12.3% (*P* ≤ 0.001) and 9.8% (*P* ≤ 0.01), respectively, in H23 cells by 8 hours, and from 8.5% (mock control) to 19.8% (*P* ≤ 0.01) and 19.3% (*P* ≤ 0.01), respectively, in H460 cells by 4 hours (Supplementary Figure S4A). The trend persisted in H460 cells at 8 hours, however the difference between mock control and combination treatment was no longer significant. We also observed no significant differences in the overall percentage of cells that incorporated EdU at either time point in either cell line (Supplementary Figure S4B).

### The combination of AZD6738 and cisplatin induces rapid cell death in ATM-deficient NSCLC cells

**Figure 4:**
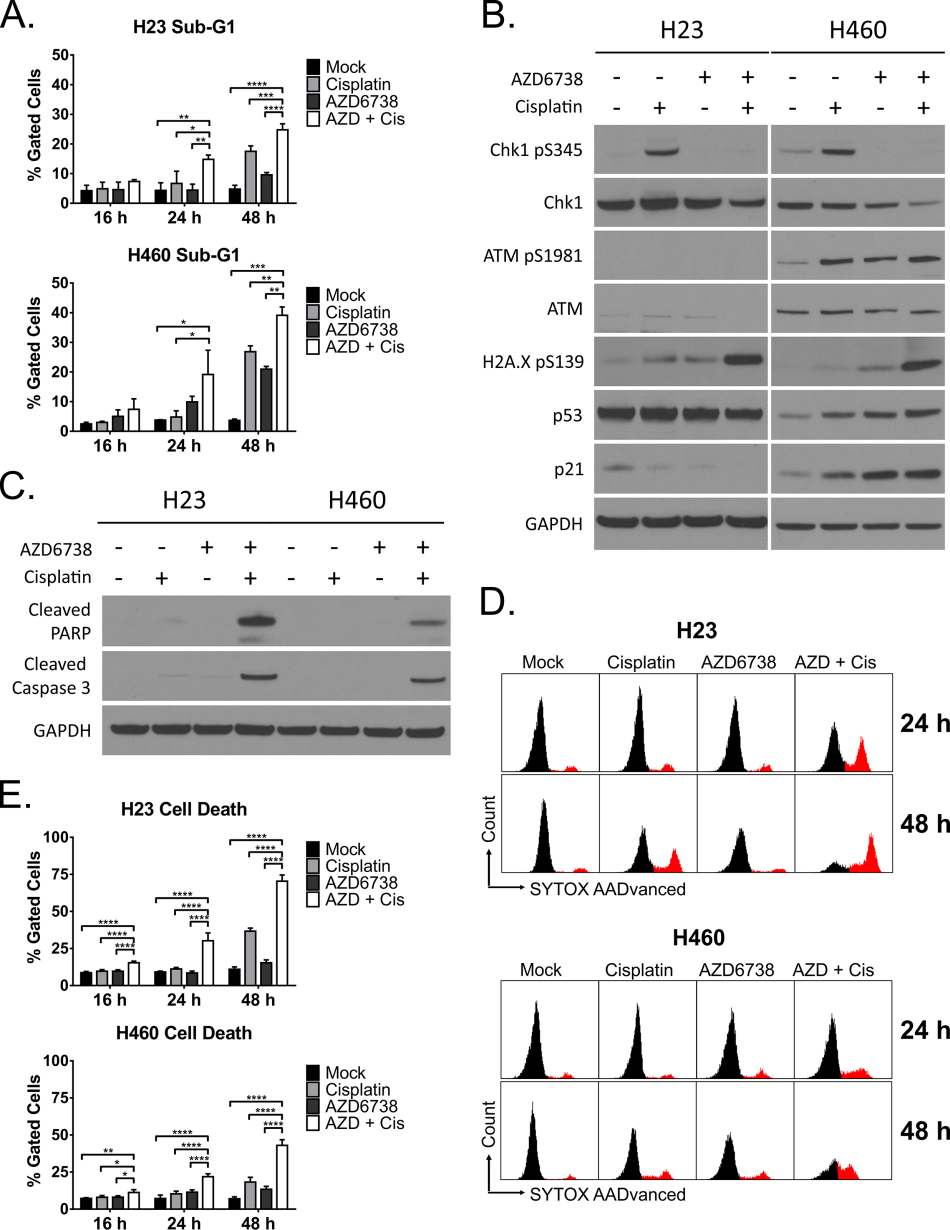
The combination of AZD6738 and cisplatin causes dramatic cell death of ATM-deficient cells independent of the ATM-p53 signaling pathway H23 and H460 cells were treated with 1.0 μM AZD6738, 5 μM (H23) or 1.67 μM (H460) cisplatin, combination, or mock control. **A.** Cell death assessed by analysis of the sub-G1 population following 16, 24, and 48 hour treatment. Bars represent the mean percentage of gated cells (± SD). Data averaged from 2 independent experiments, each with 1–2 replicates per condition. Statistical significance by ANOVA with Tukey's multiple comparison test denoted for AZD + Cis compared to other treatments as follows: *P ≤ 0.05, **P ≤ 0.01, ***P ≤ 0.001, ****P ≤ 0.0001. **B–C.** Western blots following 24 hour treatment with AZD6738, cisplatin, combination, or mock. B. Blots for phosphorylation of Chk1 (S345), ATM (S1981), and H2A.X (S139), and induction of p53 and p21. C. Blots depicting cleavage of caspase-3 and PARP. **D–E.** Cell death assessed by DNA dye (SYTOX AADvanced) exclusion following 16, 24 and 48 hour treatment. D. Representative histograms depicting the population of cells that stained positive for the DNA dye following 16, 24, or 48 hour treatment with AZD6738, cisplatin, combination, or mock. E. Quantification of cell death. Bars represent the mean percentage of gated cells (± SD) that stained positive. Data averaged from 2 independent experiments, each with 2 replicates per condition (*n* = 4 total). Statistical significance by ANOVA with Tukey's multiple comparison test denoted for AZD + Cis compared to other treatments as follows: *P ≤ 0.05, **P ≤ 0.01, ***P ≤ 0.001, ****P ≤ 0.0001.

The presence of a sub-G1 population is indicative of DNA degradation during cell death via apoptosis [43]. We quantified the percentage of cells in sub-G1 from our 16 and 24 hour cell cycle experiments, as well as assessed sub-G1 content after 48 hour treatment (Figure 4A). The combination of AZD6738 and cisplatin caused a significant increase in cell death by 24 hours in both H23 (14.8% vs. 4.3% for mock, *P* ≤ 0.01) and H460 (19.1% vs. 3.8% for mock, *P* ≤ 0.05, compared to mock) cells. By 48 hours, the percentage of cells in sub-G1 further increased in both H23 (24.8% vs. 4.8% for mock, *P* ≤ 0.0001) and H460 (39.1% vs. 3.7% for mock, *P* ≤ 0.01). Cisplatin alone also induced significant cell death by this time point (17.5%, *P* ≤ 0.0001 for H23; 26.8%, *P* ≤ 0.001 for H460).

Our sub-G1 data indicated greater cell death in H460 cells. To better understand if this was driven by a DNA damage response induced apoptosis, we examined signaling through the ATM-p53 pathway, as well as examined caspase-3 and PARP cleavage, in both cell lines (Figure 4B–4C). In both H23 and H460 cells, AZD6738 abrogated cisplatin-induced Chk1 phosphorylation. In H460 cells, treatment with AZD6738, cisplatin, and combination all resulted in activation of ATM (S1981), stabilization of p53, and induction of p21 (Figure 4B). Combination treatment caused the greatest effects on this pathway, as well as a marked increase in H2A.X phosphorylation (S139) not observed with AZD6738 or cisplatin alone. ATM-deficient H23 cells also exhibited a similar increase in phospho-H2A.X independent of the ATM-p53 pathway. To further validate ATM deficiency in H23 cells, we examined Chk2 phosphorylation (T68). While cisplatin treatment induced Chk2 phosphorylation, this was abrogated by AZD6738 in H23 cells but not H460 cells, indicating ATR dependent activation of Chk2 in the absence of active ATM (Supplementary Figure S5A). Caspase-3 and PARP cleavage increased dramatically in both cell lines following treatment with the combination of AZD6738 and cisplatin (Figure 4C). In agreement with the sub-G1 data, this suggested induction of apoptosis following treatment with the combination.

In contrast to the sub-G1 data, our cell viability studies indicated a more dramatic decrease in viability following treatment with AZD6738 and cisplatin in ATM-deficient H23 cells compared to H460 cells. We hypothesized that in addition to apoptosis, H23 cells may undergo non-apoptotic cell death. To determine overall cell death following treatment of these cell lines with AZD6738, cisplatin, or combination, we performed DNA dye (SYTOX AADvanced) exclusion assays at 4, 8, 16, 24, and 48 hours. We observed no significant increases in cell death in either cell line at 4 or 8 hours (Supplementary Figure S5B). In H23 cells treated with the combination, we noted a dramatic increase in cell death at 24 hours (30.2% vs. 9.2%, *P* ≤ 0.0001) and 48 hours (70.4% vs. 11.0%, *P* ≤ 0.0001) compared to mock control cells (Figure 4D–4E). This extent of cell death was substantially greater than observed in our sub-G1 analyses. Conversely, increases in cell death in H460 cells treated with the combination were similar to those observed in our sub-G1 analyses, with 21.9% death (vs. 7.2% in mock, *P* ≤ 0.0001) at 24 hours and 43.0% (vs. 7.0% in mock, *P* ≤ 0.0001) at 48 hours (Figure 4D–4E).

### Knockdown of ATM sensitizes NSCLC cells to the combination of AZD6738 and cisplatin *in vitro*

**Figure 5:**
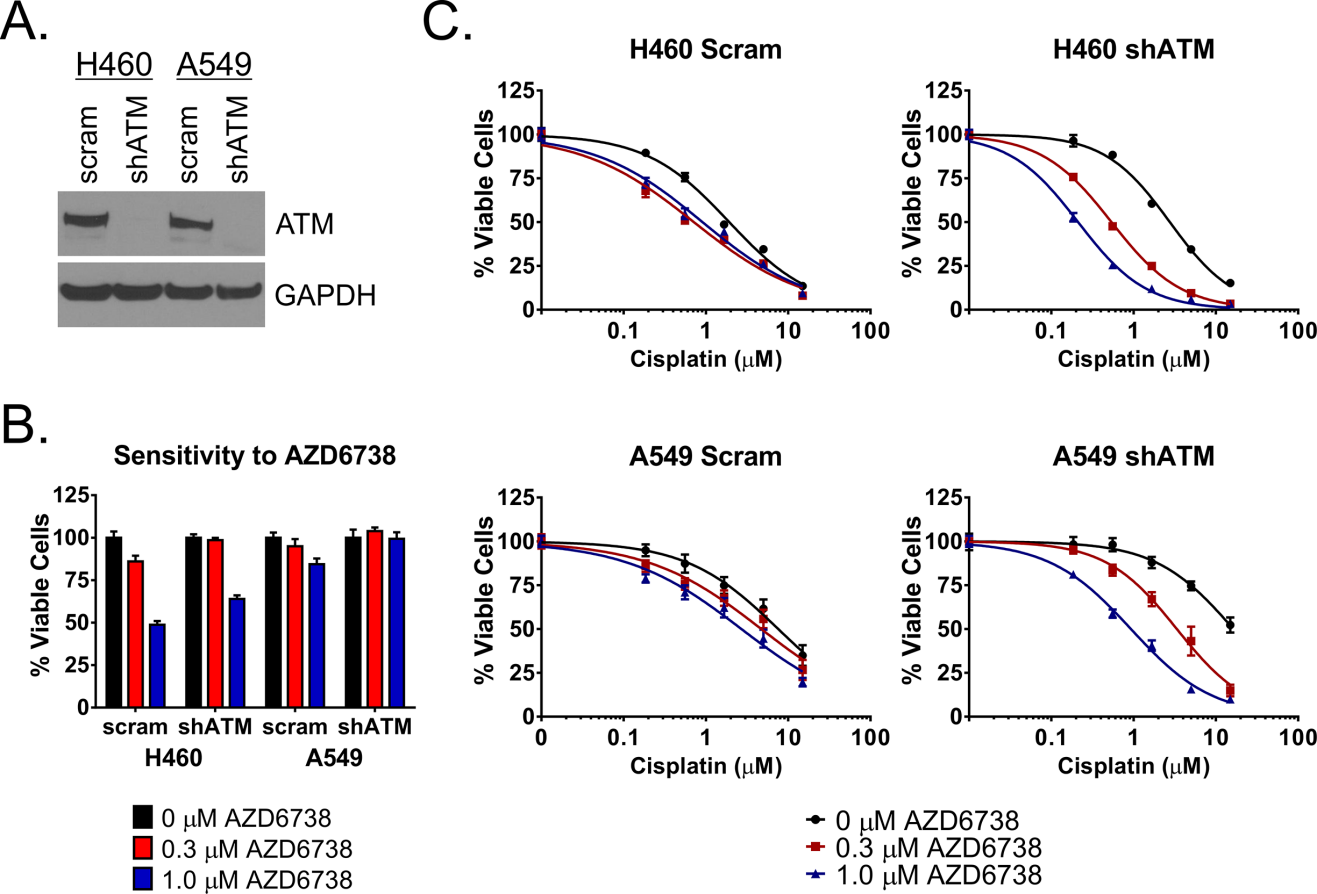
AZD6738 sensitizes ATM knockdown cells to cisplatin **A.** Western blot depicting successful knockdown of ATM protein in shATM H460 and A549 cells compared to scrambled control. **B.** Relative sensitivity of shATM and scrambled control cells to AZD6738. Bars represent the mean percentage of viable cells (± SD) relative to the mean of untreated control cells. Data averaged from 2 independent experiments, each with 3 replicates per condition (*n* = 6 total). **C.** Shift in cisplatin sensitivity in H460 and A549 ATM knockdown cell lines compared to scrambled control lines. Curves represent the mean percentage of viable cells (± SD) relative to the mean of 0 μM cisplatin controls within each AZD6738 treatment condition. Data averaged from 2 independent experiments, each with 3 replicates per condition (*n* = 6 total).

To determine the role for ATM deficiency in sensitivity of NSCLC cells to the combination of AZD6738 and cisplatin, we knocked down ATM protein in H460 and A549 cells using a short hairpin RNA construct (shATM) (Figure 5A). We then performed curve shift analysis by treating shATM and scrambled control cell lines with a fixed 0.3 or 1.0 μM dose of AZD6738 in combination with cisplatin (0.19–15.0 μM) and assessing cell viability after 48 hour treatment. Intriguingly, we noted that the shATM cell lines exhibited decreased sensitivity to AZD6738 alone (Figure 5B) as well as cisplatin alone in this assay (Figure 5C and Table 1). Despite this, knockdown of ATM sensitized both H460 and A549 cells to the combination of AZD6738 and cisplatin (Figure 5C and Table 1). In H460 shATM cells, 1.0 μM AZD6738 shifted cisplatin IC_50_ from 2.77 μM to 0.22 μM (12.59-fold), compared to 2.36 μM to 0.43 μM (5.44-fold) and 1.83 uM to 0.84 (2.18-fold) in wildtype and scrambled control cells, respectively. Similar results were seen in A549 shATM cells. Treatment with 1.0 μM AZD6738 reduced cisplatin IC_50_ from 19.84 μM to 1.02 μM (19.45-fold) versus reductions from 7.79 μM to 2.61 μM (2.98-fold) and 9.68 μM to 3.48 μM (2.78- fold) in wildtype and scrambled control cells, respectively. Treatment with 0.3 μM AZD6738 also resulted in greater shifts in cisplatin IC_50_ and lower net IC_50_ values in shATM cell lines compared to wildtype and scrambled control lines (Table 1).

**Table 1:** Effect of AZD6738 on cisplatin sensitivity in wildtype, scrambled control, and ATM knockdown H460 and A549 cell lines

	H460			A549		
	Wt	Scram	shATM	Wt	Scram	shATM
**Cisplatin IC_50_ (μM)**	2.36	1.83	2.77	7.79	9.68	19.84
**0.3 μM AZD + Cisplatin**	0.72	0.66	0.55	4.68	6.40	4.18
**Fold Shift 0.3 μM AZD**	3.29	2.77	5.04	1.66	1.51	4.75
**1.0 μM AZD + Cisplatin**	0.43	0.84	0.22	2.61	3.48	1.02
**Fold Shift 1.0 μM AZD**	5.44	2.18	12.59	2.98	2.78	19.45

Values represent the mean IC_50_ for cisplatin alone and in combination with 0.3 or 1.0 μM AZD6738 and the fold shift in IC_50_ resulting from the addition of AZD6738. Values calculated from the average of normalized data from 2 independent experiments, each with 3 replicates per condition (*n* = 6 total).

### The combination of AZD6738 and cisplatin has efficacy in NSCLC xenograft models and causes rapid regression of ATM-deficient NSCLC tumors

Next we assessed the efficacy of AZD6738 alone and in combination with cisplatin *in vivo*. Effects on food consumption and body weight are dose limiting for AZD6738 in mice, rats and dogs, and are typically accompanied by atrophic/degenerative histopathology in the gastrointestinal tract after repeated dosing (AstraZeneca, personal communication). AZD6738 caused hypocellularity in multiple lymphoid tissues and bone marrow toxicity correlated with a decrease in all cell lineages in the peripheral blood. There was a minimal increase in alveolar macrophages. Recovery of these effects was seen after cessation of dosing.

**Figure 6:**
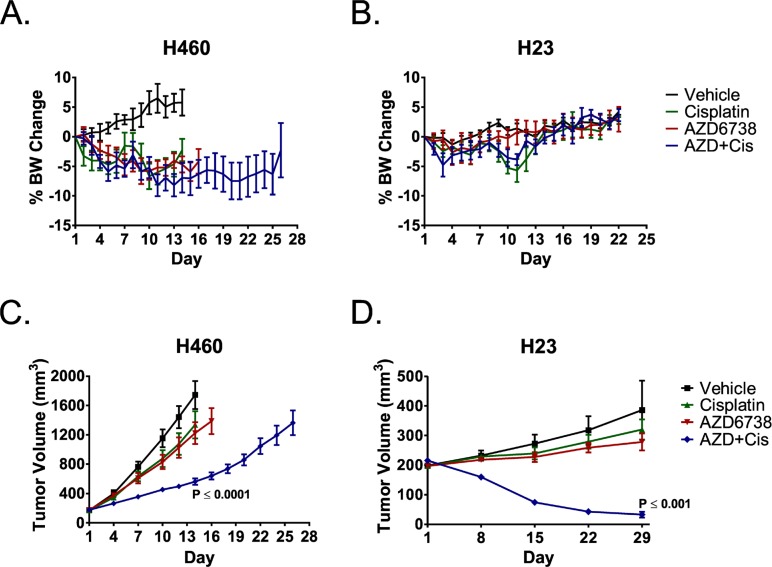
AZD6738 potentiates cisplatin efficacy in NSCLC xenografts, and the combination causes rapid regression of ATM-deficient H23 tumors Mice bearing H460 or H23 xenografts were treated with 3 mg/kg cisplatin (IP, q7d x 2), 50 mg/kg (H460) or 25 mg/kg (H23) AZD6738 (PO, qd x 14), the combination of cisplatin and AZD6738, or vehicle. **A–B.** Curves depicting mean% change in body weight (± SEM) for H460 (A) and H23 (B) bearing mice. **C–D.** Tumor growth curves depicting response of H460 (C) and ATM-deficient H23 (D) tumors to treatment. Curves represent mean tumor volume (± SEM). Mice per treatment arm: H460, *n* = 6 (Vehicle) or *n* = 7 (remaining arms); H23, *n* = 6. Statistical significance (compared to Vehicle) determined by ANOVA with Tukey's multiple comparison test.

We treated nude mice bearing H460 tumors with 50 mg/kg AZD6738 (PO) and mice bearing ATM- deficient H23 tumors with 25 mg/kg AZD6738 (PO) and for 14 consecutive days. Mice received 3 mg/kg cisplatin (IP) on days 1 and 8 of the two week treatment cycle. Body weight loss was the dose limiting toxicity with daily administration of 50 mg/kg AZD6738, alone and in combination with cisplatin. However, body weights remained within protocol guidelines for the duration of treatment, and no animal on study lost greater than 14.3% BW at any point during treatment (Figure 6A). Conversely, 25 mg/kg AZD6738 was well tolerated, with mean body weight (BW) losses of less than 2.7% and 4.8% in the single agent and combination arms, respectively (Figure 6B). Mice treated with the combination exhibited BW loss similar to those that received cisplatin alone.

The combination of 50 mg/kg AZD6738 and cisplatin resulted in a 75.5% mean tumor growth inhibition (TGI) of H460 xenografts at day 14 (*P* ≤ 0.0001 compared to vehicle) (Figure 6C). Growth of tumors treated with the combination was also significantly different from that of tumors treated with cisplatin or AZD6738 alone (*P* ≤ 0.01 or *P* ≤ 0.05, respectively). Growth delay for the combination treatment was 12 days (day 26 vs. day 14), although only one of seven tumors had reached the 2000 mm^3^ endpoint on day 26. While modest growth inhibition was observed in the single agent AZD6738 and cisplatin treatment arms, the differences in growth were not statistically significant (*P* ≥ 0.05).

Strikingly, the combination of 25 mg/kg AZD6738 and cisplatin resulted in rapid and near complete tumor regression (84.8%) of ATM-deficient H23 tumors by day 29 (Figure 6D). The mean change in tumor growth was significantly different than that of the mock, cisplatin, and AZD6738 treatment arms (*P* ≤ 0.001, *P* ≤ 0.01, and *P* ≤ 0.05, respectively). Treatment with cisplatin or AZD6738 alone did not result in significant inhibition of tumor growth (*P* > 0.05). After day 29, mice in the combination treatment arm were observed weekly for tumor regrowth. Of the six mice that received combination treatment, three exhibited complete tumor resolution by days 43, 64, and 92, respectively. There was no visual or palpable evidence of tumor out to a final observation on day 113. In the remaining three mice, tumors began to slowly regrow within 3–5 weeks of the end of treatment.

We confirmed by immunohistochemistry that 25 mg/kg AZD6738 inhibits ATR activity in H23 xenografts. Mice were treated with 25 mg/kg AZD6738 daily for 8 consecutive days, 3 mg/kg cisplatin on days 1 and 8, combination, or vehicle, and tumors were harvested six hours following the final dose on day 8. Tumors from mice treated with AZD6738 exhibited reduced phosphorylation of T1989 (Supplementary Figure S6), a marker of active ATR [44, 45].

## DISCUSSION

AZD6738 is a highly selective and potent inhibitor of ATR kinase activity that is both orally active and bioavailable. As previously described for ETP-46464, and the selective ATR kinase inhibitors VE-821 and VX-970 (VE-822) [21–26, 32, 33], AZD6738 induces ATM kinase-dependent DNA damage signaling and potentiates cell killing by cisplatin.

To our knowledge, this is the first documentation that ATR kinase inhibition can induce senescence in cancer cells. This was anticipated from the finding that inhibition of ATR kinase induced by an inactivating mutation in the ATR activation domain (AAD) of TOPBP1, an allosteric activator of ATR kinase activity, caused cellular senescence in mouse embryonic fibroblasts [35]. Since ATR signaling can also drive cells into senescence in the absence of DNA breaks [46], ATR kinase signaling appears to be a key mediator of senescence. AZD6738 induced senescence in *Kras* mutant, p53-wildtype A549 and H460 cells, consistent with activation of the ATM-p53-p21 signaling pathway as well as induction of p27. However, AZD6738 did not induce senescence in *Kras* mutant H23 cells which lack a functional ATM-p53 axis. The senescence rather than apoptosis of A549 cells observed following AZD6738 treatment is consistent with a prior report that A549 cells possess an “apoptosis-reluctant” phenotype [47]. Conversely, AZD6738 treatment elicited an apoptotic response H460 cells, in addition to senescence, and our data suggests that cell death predominates over senescence in this cell line. Given that H460 cells overexpress c-myc, our observations are consistent with the finding that ATR is required to limit apoptosis associated with Myc-induced replication stress [48].

AZD6738 potentiates the cytotoxicity of cisplatin and gemcitabine in the four NSCLC cell lines we tested. Notably, AZD6738 strongly synergizes with cisplatin in an ATM-deficient NSCLC cell line, as has been reported with other ATR kinase inhibitors and other cancer cells previously [23]. In both ATM-deficient H23 cells and ATM-wildtype H460 cells, AZD6738 causes cell cycle aberrations, with cells accumulating at the G1/S border and in early S-phase. Both cell lines exhibit an apoptotic response following treatment with the combination of AZD6738 and cisplatin, evident by caspase-3 and PARP cleavage. However, while H460 cells with activated ATM signaling appear to undergo apoptosis, ATM- deficient H23 cells exhibit considerably greater cell death in dye exclusion studies than observed in sub-G1 analyses, suggesting that an alternative mode of cell death predominates in this context.

In addition to the synergy of AZD6738 with cisplatin observed in ATM-deficient H23 cells, shRNA knockdown of ATM resulted in similar synergy with cisplatin *in vitro* in two p53-wildtype NSCLC cell lines. ATM mutations are found in lymphoid tumors [49–51], pancreatic cancers [52], as well as 7% of lung adenocarcinomas [53]. These data have important clinical implications as they suggest that AZD6738 has the potential to enhance the efficacy-of-standard of care treatments in > 150,000 NSCLC patients in the USA each year, particularly those harboring ATM-deficient tumors.

ATR is an essential protein in mice and mammalian cells [15–18], and there is a pervasive view that ATR kinase inhibitors will not be tolerated in the clinic. Here, while body weight loss was dose limiting with daily administration of 50 mg/kg AZD6738 by oral gavage for 14 consecutive days, treatment with 25 mg/kg AZD6738 daily for 14 consecutive days was tolerated in mice, as food consumption and body weight were relatively unchanged. This dose of AZD6738 was shown to inhibit ATR in mice and the expected plasma half-life of AZD6738 in mice is approximately 6 hours (Astra Zeneca, personal communication). We show that treatment with 25 mg/kg AZD6738 daily for 8 days, inhibits ATR activity in H23 xenografts, evident by reduced immunohistochemical staining for phospho-ATR T1989. Furthermore, AZD6738 clearly has activity at this dose as the H23 xenografts strongly responded to the combination of AZD6738 and cisplatin, but showed no response to cisplatin alone. In other studies, AZD6738 has been successfully administered to mice at 25 mg/kg PO twice daily (13 days) and at 75 mg/kg PO once daily for 21 days (AstraZeneca, personal communication).

ATR kinase signaling is frequently high in cancer cells and this is thought to be associated with replication stress, making ATR an intriguing target [54–56]. Inhibition of ATR with AZD6738 enhances the efficacy of the first-line therapeutic agent cisplatin both *in vitro* and *in vivo*, and the standard-of-care agent gemcitabine *in vitro*. AZD6738 is orally active and bioavailable and is well tolerated in mice. Importantly, AZD6738 strongly synergizes with cisplatin in ATM-deficient models of NSCLC, and causes near complete tumor regression in an ATM-deficient xenograft model. Taken together, these data support the potential clinical utility of AZD6738 for the treatment of NSCLC.

## MATERIALS AND METHODS

### Cell lines

NCI-H23, NCI-H460, A549, NCI-H358, and IMR-90 were purchased from the American Type Culture Collection (ATCC) in November, 2013. Cells were periodically tested for mycoplasma (Lonza MycoAlert Mycoplasma Detection Kit). Experiments were conducted on cells with fewer than 20 passages after initial resuscitation. Cells were cultured in RPMI-1640 (containing 2 mM l-glutamine) supplemented with 10% FBS, penicillin/streptomycin in a humidified incubator at 37°C with 5% CO_2_. Cells were seeded approximately 20–22 h prior to treatment with AZD6738 or chemotherapeutics.

### Drugs and reagents

AZD6738 was provided by AstraZeneca. For *in vitro* use, AZD6738 was dissolved in DMSO at 30 mM and diluted in DMSO to desired working concentrations. The final DMSO concentration in media for all conditions and controls was 0.1% for AZD6738 dose response experiments, 0.05% for AZD6738 + chemotherapy viability experiments, and 0.025% for all experiments involving 0.3 μM and 1.0 μM doses of AZD6738. For *in vivo* use, AZD6738 was dissolved in DMSO at a concentration of 25 mg/mL or 50 mg/mL and diluted 1:5 in propylene glycol. An equal volume of H_2_O was added to yield 2.5 mg/mL or 5 mg/mL AZD6738 in 10% DMSO, 40% propylene glycol, and 50% sterile dH_2_O. Cisplatin (APP Pharmaceuticals, Inc.) and gemcitabine (Sagent Pharmaceuticals) were purchased from the University of Pittsburgh Cancer Institute pharmacy. Gemcitabine was dissolved in sterile saline (0.9% sodium chloride) according to the manufacturer's instructions. Both agents were diluted in sterile 1x PBS for *in vitro* use. Cisplatin was diluted in sterile saline for *in vivo* use. Docetaxel (LC Labs) was kindly provided by the lab of Dr. Timothy F. Burns (University of Pittsburgh) and dissolved in DMSO.

### Cell viability assays

Cells were treated in white walled, clear bottom 96-well plates with the indicated doses of AZD6738, cisplatin, gemcitabine, or combination for 48 h. ATP levels were assessed as surrogate measure of viability was assessed using the CellTiter-Glo Luminescent Cell Viability Assay (Promega) and Safire^2^ plate reader (Tecan). Raw data were corrected for background luminescence prior to further analysis. For AZD6738 treatment, log dose response curves were generated in GraphPad Prism 6 by nonlinear regression (log(inhibitor) vs. response with variable slope) of log-transformed (x = log(x)) data normalized to the mean of untreated controls. GI_50_ values, defined as the dose X at which Y = 50%, were extrapolated from dose response curves. For combination treatments, data were normalized to the mean of untreated controls. Loewe excess matrices were generated using Chalice Analyzer Online (Horizon CombinatoRx) and mean normalized inhibition values. For AZD6738 + cisplatin curve shift experiments, data were normalized to the mean of 0 μM cisplatin controls within each AZD6738 treatment condition. Log dose response curves were generated in GraphPad Prism 6 by nonlinear regression (log(inhibitor) vs. normalized response with variable slope) of log-transformed (x = log(x)), normalized data. IC_50_ values were calculated by Prism 6.

### Immunoblotting

Cells were treated with the indicated doses of AZD6738, cisplatin, combination, or mock for 24 h. Protein lysates were generated by scraping adherent cells in lysis buffer (150 mM NaCl, 50 mM Tris-HCL, 5 mM NaF, 1% Tween 20, 0.5% IGEPAL CA-630, protease inhibitor cocktail, pH 7.5) and incubating on ice for 30 min. For AZD6738 + cisplatin experiments, detached cells were pelleted from the media and combined with the adherent cell lysate. SDS-PAGE using 4–12% Bis-Tris gels (NuPAGE Novex) and Western blotting were performed using standard techniques. Antibody details are provided in the supplementary methods. Following detection of phospho-proteins, membranes were stripped for 25 min at room temperature in Restore stripping buffer (Thermo Scientific) and re-probed for corresponding total protein. Images of blots were acquired at 24-bit depth using a Canon LiDE110 scanner and were processed (converted to 8-bit, cropped) using ImageJ.

### Crystal violet colony formation and senescence assays

Cells were treated (in triplicate) in 12-well plates with 0.3 μM, 1 μM AZD6738, or mock for 48 h. Following treatment, AZD6738 was removed, and cells were cultured an additional 2–4 days in fresh media. Colony formation was visualized by staining with 0.5% crystal violet in 95% EtOH. Images were captured with an Olympus SZX10 stereo microscope and DP26 camera. Unprocessed images were resized for inclusion in figures. Experiments were repeated at least three times to ensure consistent results. Senescence-associated β-galactosidase activity was assessed using the Biovision Senescence Detection Kit. Images were acquired using a Leica DMI3000B inverted microscope (20X objective) and DFC420C camera. Unprocessed images were resized for inclusion in figures.

### Replating assays for long term cell viability

Cells were treated with 0.3 μM, 1 μM AZD6738, or mock for 48 h. Following treatment, cells were seeded in 96 well plates (4 replicates) at equal density per condition and grown for an additional 6 days. Viability was assessed on day 8 using the CellTiter-Glo Luminescent Cell Viability Assay (Promega) and Safire^2^ plate reader (Tecan). Background corrected data were normalized to the mean of untreated controls.

### Flow cytometry

Cells were treated with AZD6738, cisplatin, combination, or mock for the indicated times and collected following trypsinization. For cell cycle and cell death (DNA dye exclusion) experiments, the existing media and 1x PBS wash were retained and added to collected cells. Data collection and analyses were performed using an Accuri C6 flow cytometer (BD Biosciences). Samples were gated on SSC-A vs. FSC-A followed by SSC-H vs. SSC-A for removal of doublets. Approximately 10,000 gated events were collected. For cell cycle analysis, collected samples were washed with 1% FBS/1x PBS and fixed in 70% EtOH at 4°C for at least 24 h. Cells were then washed with 1% FBS/1x PBS, incubated for 5 min at room temperature in phospho-citrate buffer (0.064 M sodium phosphate, 0.001 M citric acid, in 1% FBS/1x PBS), and stained with propidium iodide solution (0.05 mg/mL propidium iodide, 0.1 mg/mL RNase A, in 1% FBS/1x PBS) for 15 min at 37°C. Additional gating on FL2-H vs. FL2-A was used to ensure removal of doublets. Quantitation of the percentage of cells in each cell cycle phase was performed using FL2-A histograms. For EdU proliferation experiments, collected samples were fixed and stained using the Click-iT Plus EdU Alexa Fluor 488 Flow Cytometry Assay Kit (Life Technologies) according to the manufacturer's instructions. DNA content was stained with FxCycle PI/RNase Staining Solution (Life Technologies). Quantitation of EdU incorporation was performed using dot plots of FL1-A vs. FL2-A. For cell death analysis via DNA dye exclusion, collected samples were washed with 1x PBS and stained with 1 μM SYTOX AADvanced dead cell stain (Life Technologies) in 1x PBS for 5–10 min at room temperature, and placed on ice for immediate analysis. Cell death was quantified using FL3-A vs. SSC-A dot plots and displayed via FL3-A histograms.

### shRNA knockdown of ATM

Lentiviral particles containing short hairpin RNA (shRNA) oligonucleotides were generated in 293T cells using the pLKO.1 TRC cloning vector (Addgene). Lentivirus production and infection of H460 and A549 cells were performed according to The RNAi Consortium (TRC) Library Production and Performance Protocols (Broad Institute) [57]. ATM shRNA construct #54 was obtained from TRC. Selection with 1.0 μg/mL puromycin began 24 h after infection.

### Mouse xenograft experiments

Protocols for animal experiments were approved by the University of Pittsburgh Animal Care and Use Committee and were strictly followed. Female athymic nude (*Foxn1*^nu^) mice, 6–7 weeks old, were purchased from Harlan Laboratories. H23 (3 × 10^6^ cells) or H460 (7 × 10^5^ cells) were injected subcutaneously into the right hind flank in a volume of 100 μL (equal parts 1x PBS and Matrigel). Cells were tested for mycoplasma (Lonza MycoAlert Mycoplasma Detection Kit) prior to inoculation in mice. Mice began receiving treatment once tumors reached approximately 220 mm^3^ (± 15%) for H23 or 180 mm^3^ (± 15%) for H460. Tumor volume was calculated as (L × W^2^)/2. AZD6738 was administered by oral gavage (qd × 14) at 25 mg/kg (H23) or 50 mg/kg (H460). Cisplatin was administered intraperitoneally (q7d × 2) at 3 mg/kg. The dosing volume was 10 mL/kg. Growth curves depict mean (± SEM) tumor volume over time. Mean tumor growth inhibition was calculated as TGI = (1–(T_f_–T_0_)/(C_f_–C_0_))*100, where T_f_ and T_0_ represent final and initial mean tumor volumes in the treatment arm, respectively, and C_f_ and C_0_ represent final and initial mean tumor volumes in the vehicle control arm, respectively. Mean tumor regression was calculated as % Regression = ((T_0_–T_f_)/T_0_)*100. For H460 xenografts, the experimental endpoint was defined as the day on which any single tumor within the treatment arm reached 2000 mm^3^. Tumor growth delay is defined as the difference in the number of days to reach the endpoint for a given treatment arm compared to vehicle control.

### Statistical analyses

ANOVA with Dunnett's and Tukey's multiple comparison tests (95% confidence interval) were performed in Graphpad Prism 6. Statistical analysis of xenograft data was performed by comparing mean growth (measured as change from baseline tumor volume to tumor volume at day 14 for H460 or day 29 for H23).

## SUPPLEMENTARY MATERIAL TABLES AND FIGURES


