# Supplementary material for: The orally active and bioavailable ATR kinase inhibitor AZD6738 potentiates the anti-tumor effects of cisplatin to resolve ATM-deficient non-small cell lung cancer in vivo
Source: Oncotarget. 2015 Oct 27;6(42):44289–305. doi: 10.18632/oncotarget.6247 (PMC4792557; doi:10.18632/oncotarget.6247)
Supplement: Supplementary file 1 [file oncotarget-06-44289-s001.pdf]

The orally active and bioavailable ATR kinase inhibitor AZD6738 potentiates the anti-tumor effects of cisplatin to resolve ATM-deficient non-small cell lung cancer *in vivo*

Supplementary Materials

Synergy matrix assays

Cells were seeded in white walled, clear bottom 96-well plates. Cells were treated for 48 hours with AZD6738 (0.02–15 µM) and cisplatin (0.02–15 µM), gemcitabine (1.4–1000 nM), or docetaxel (0.14–100 nM) in a two dimensional dosing matrix with 3-fold serial dilutions along each axis. Immediately following treatment, viability was assessed using the CellTiter-Glo Luminescent Cell Viability Assay (Promega) and Safire^2 plate reader (Tecan). Raw data were corrected for background luminescence, and the percent inhibition of viability relative to untreated control well was calculated. Data were then analyzed using Chalice Analyzer Online (Horizon CombinatoRx).

Antibodies for immunoblotting

ATM pS1981 (ab81292) and GAPDH (ab8245) antibodies were purchased from Abcam and used at 1:2000 and 1:40000 dilution, respectively. ATR (#13934), Chk1 (#2360), Chk1 pS345 (#2348), Chk2 (#3440), Chk2 pT68 (#2661), cleaved Caspase-3 Asp175 (#9664), cleaved PARP (#5625), and H2A.X pS139 (#9718) were purchased from Cell Signaling Technology and used at 1:500 (Chk2 pT68, cleaved caspase-3, and H2A.X pS139) or 1:1000 (all others) dilution. ATM (A1106) antibody was purchased from Sigma and used at a 1:2000 dilution. p21 antibody (OP64) was purchased from EMD Millipore and used at a 1:250 dilution. p27 (sc-1641) and p53 (sc-126) antibodies were purchased from Santa Cruz Biotechnology and used at a 1:250 and 1:500 dilution, respectively. Anti-rabbit (Rockland, 611–1322) and anti-mouse (Jackson ImmunoResearch Laboratories, 315–035–045) HRP-conjugated secondary antibodies were used at 1:10000 and 1:5000 dilution, respectively. For panels of blots, GAPDH loading control bands shown are representative and were confirmed across multiple blots.

Immunohistochemistry

H23 xenograft bearing nude mice were treated with AZD6738 (25 mg/kg, qd x8), cisplatin (3 mg/kg, days 1 and 8), combination, or vehicle. Six hours following the final dose of treatment, tumors were harvested and fixed in 10% buffered formalin phosphate prior to embedding in paraffin. Immunohistochemistry was performed on five micron sections using standard techniques and phospho-ATR S1989 antibody (GeneTex, GTX128145, 1:1000 dilution). Antigen retrieval was performed using citrate buffer. Staining was visualized using the DAB Peroxidase Substrate Kit (Vector Labs).

Supplementary Table S1: Key AZD6738 kinase selectivity data

	ATR IC_{50} cell	ATM IC_{50} cell	DNA-PK IC_{50} cell	mTOR IC_{50} cell	PI3Ka IC_{50} cell
**AZD6738 (µM)**	0.074	> 30	> 30	> 23	> 30

Supplementary Table S2: Potency of AZD6738 in NSCLC cell lines. Mean GI_{50} (± SD) (µM) averaged from 2–3 independent experiments, each with 5 replicates

	H23	H460	A549	H358
**AZD6738 GI_{50} (µM)**	2.38	1.05	5.42	6.95
**St. Dev**	0.70	0.16	0.35	0.16

Supplementary Table S3: Effect of AZD6738 on cisplatin sensitivity in H23, H460, and A549 cell lines. Values represent the fold shift in cisplatin IC_{50} resulting from the addition of 0.3 or 1.0 µM AZD6738. Values calculated from the average of normalized data from 2 independent experiments, each with 3 replicates per condition (*n* = 6 total)

	**H23**		**H460**		**A549**	
**AZD6738 (µM)**	0.3	1.0	0.3	1.0	0.3	1.0
**Fold IC_{50} Shift**	7.00	19.41	3.29	5.44	1.66	2.99

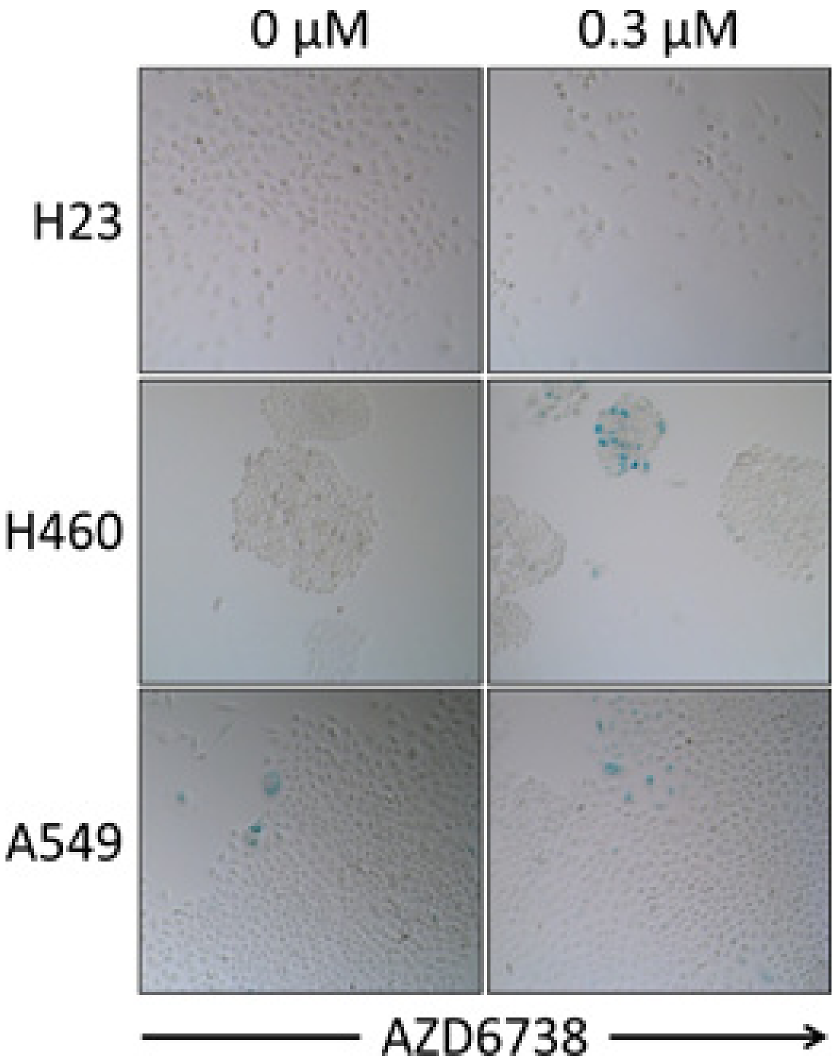

Supplementary Figure S1: Senescence associated β-galactosidase activity in NSCLC cell lines following treatment with AZD6738. H23, H460, and A549 cells were treated for 48 hours with 0.3 µM AZD6738 and incubated in drug-free media for an additional 2–3 days. Cells were then stained for senescence associated β-galactosidase activity. Representative images of SA-β-gal staining in H23 (day 5), H460 (day 4), and A549 (day 5)

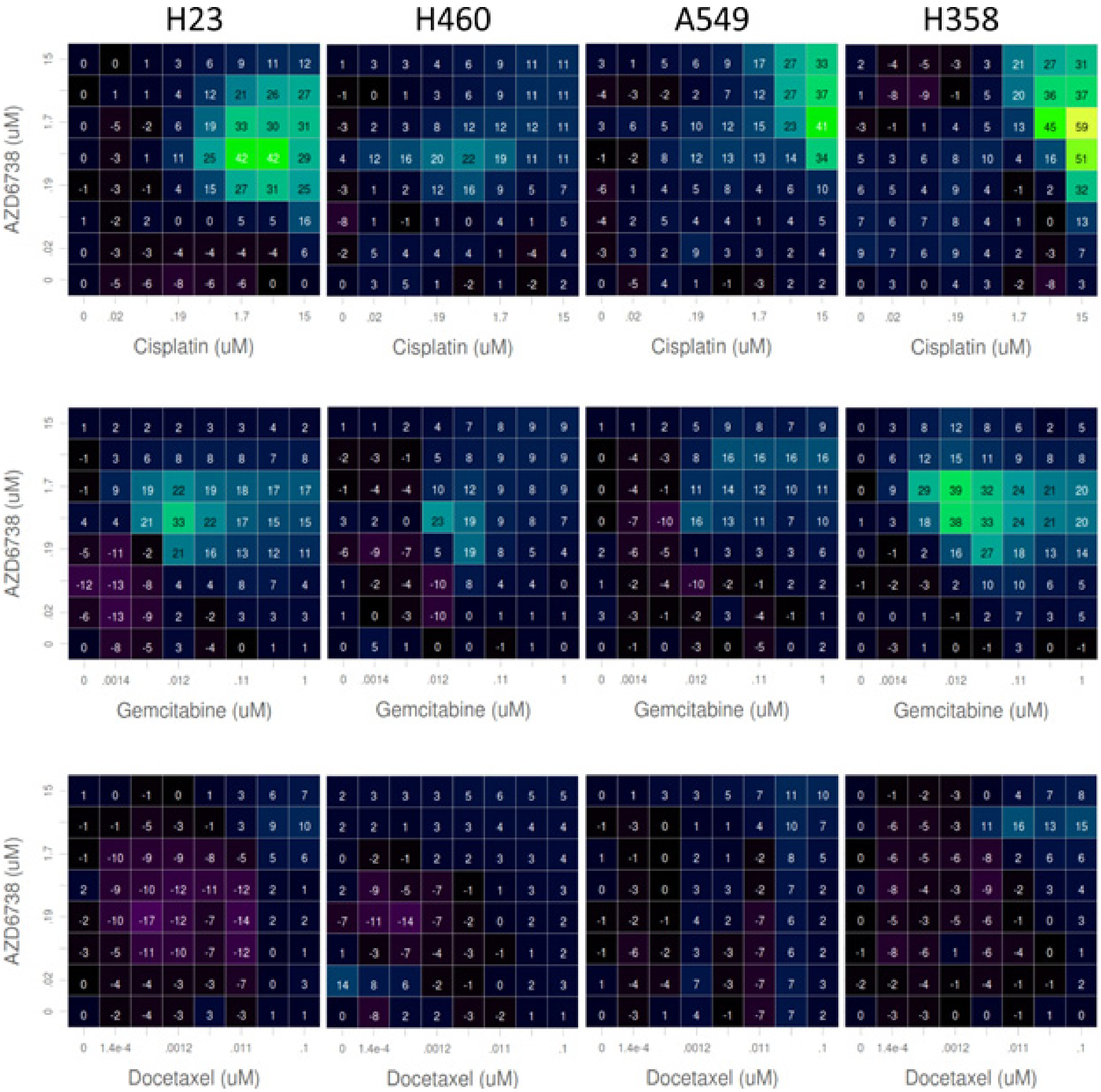

Supplementary Figure S2: AZD6738 has combinatorial activity with the DNA damaging agents cisplatin and gemcitabine in NSCLC cell lines. Cells were treated for 48 hours with AZD6738 (0.02–15 µM) and cisplatin (0.02–15 µM), gemcitabine (1.4–1000 nM), or docetaxel (0.14–100 nM) for 48 hours in a two dimensional dosing matrix with 3-fold serial dilutions along each axis. The percent inhibition of viability was assessed at 48 hour relative to the untreated control well. Color coded matrix displays the inhibition in excess of Loewe additivity, with brighter colors and corresponding higher inhibition values indicative of greater synergy.

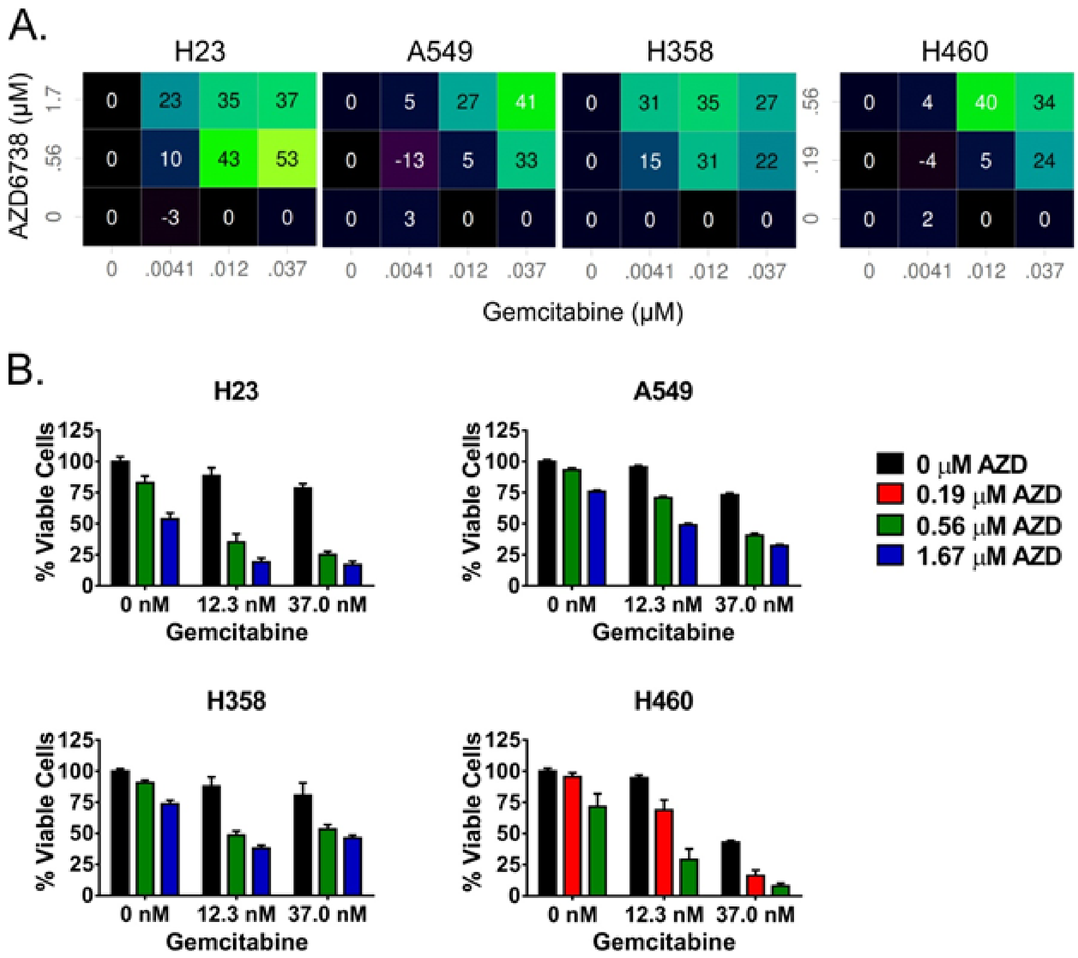

Supplementary Figure S3: AZD6738 sensitizes NSCLC cell lines to gemcitabine. Cells were treated with select doses of AZD6738 and gemcitabine for 48 hours. **A.** Color coded matrix displays the inhibition in excess of Loewe additivity, with brighter colors and corresponding higher inhibition values indicative of greater synergy. **B.** Bars represent the mean percentage of viable cells (± SD) relative to the mean of control cells, averaged from 2 (H23, A549, H358) or 3 (H460) independent experiments (each with 3–4 replicates per condition).

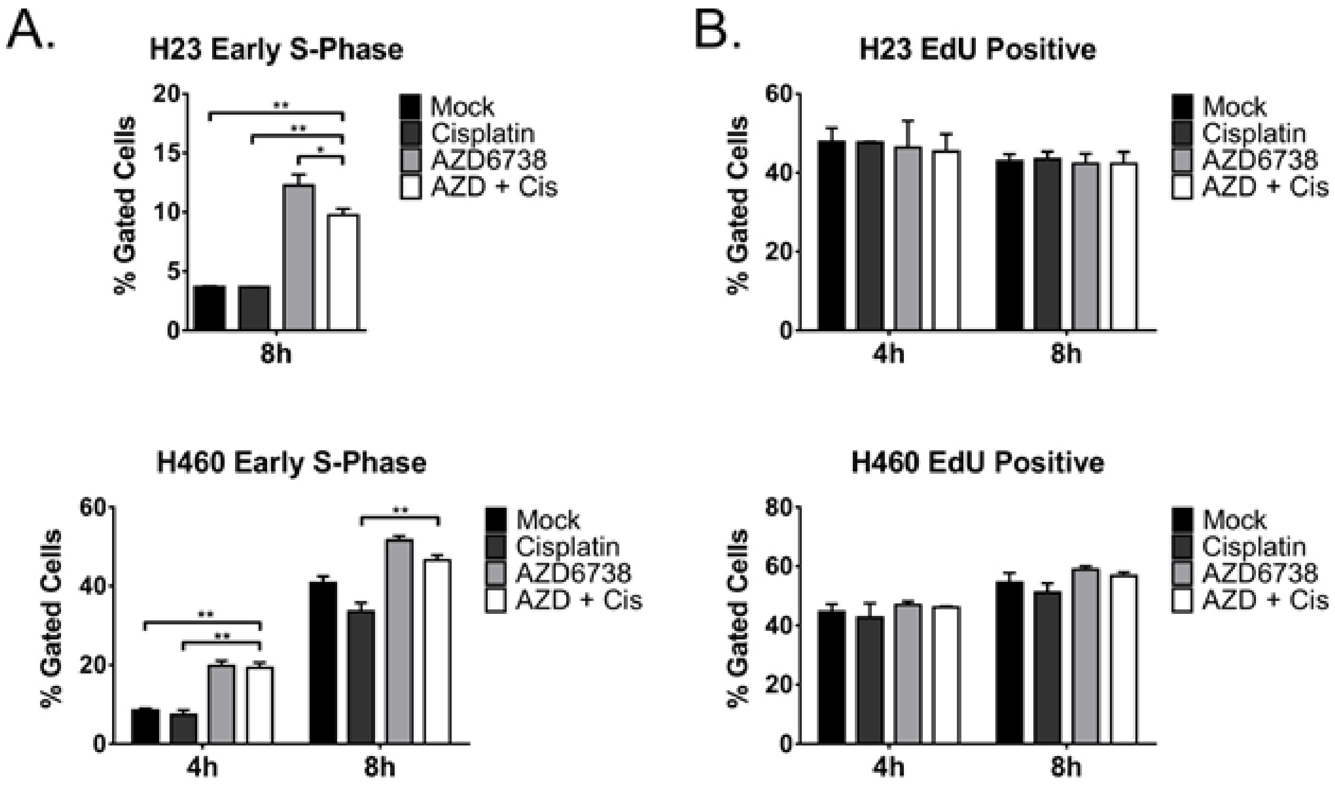

Supplementary Figure S4: AZD6738, alone and with cisplatin, causes accumulation of cells in early S-phase. A–B. H23 and H460 cells were pulsed with 10 µM EdU for 15 min (H23) or 10 min (H460) and then treated with 1.0 µM AZD6738, 5.0 µM (H23) or 1.67 µM (H460) cisplatin, combination, or mock for 4 or 8 hours. The percentages of cells in early S-phase (A) and the overall percentage of cells (B) that incorporated EdU were quantified. Bars represent the mean percentage of gated cells (± SD). Data averaged from 2 independent experiments (*n* = 2 total). Statistical significance by ANOVA with Tukey's multiple comparison test denoted for AZD + Cis compared to other treatments as follows: $^{*}P \leq 0.05$, $^{**}P \leq 0.01$.

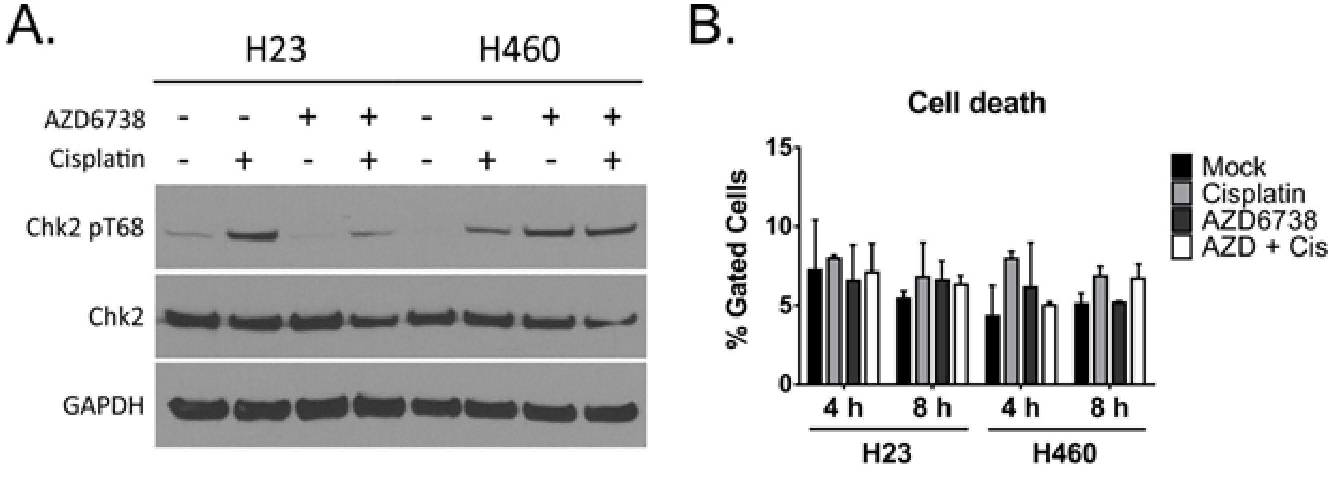

Supplementary Figure S5: The combination of AZD6738 and cisplatin does not induce cell death at early time points. A. Western blots for phospho-Chk2 (T68), total Chk2, and GAPDH loading control following 24 hour treatment of H23 and H460 cells with 1.0 µM AZD6738, 5 µM (H23) or 1.67 µM (H460) cisplatin, combination, or mock. **B.** H23 and H460 cells were treated with 1.0 µM AZD6738, 5 µM (H23) or 1.67 µM (H460) cisplatin, combination, or mock for 4 or 8 hours. Cell death was assessed by dye (SYTOX AADvanced) exclusion. Bars represent the mean percentage of gated cells (± SD) that stained positive. Data from representative experiment with 2 replicates per condition.

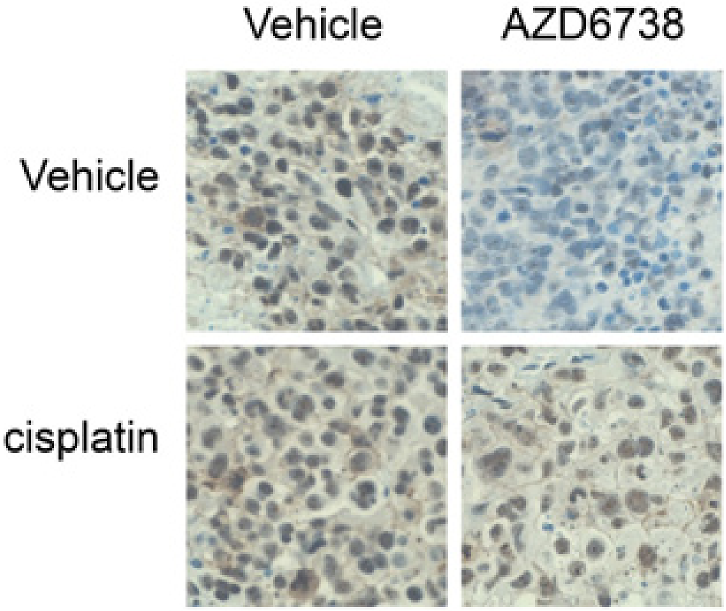

Supplementary Figure S6: AZD6738 inhibits phosphorylation of ATR (T1989) *in vivo*. Immunohistochemistry for phospho-ATR (T1989) in H23 xenograft tissues. Mice were treated with 25 mg/kg AZD6738 (PO, qd × 8), 3 mg/kg cisplatin (IP, q7d × 2), combination, or vehicle. Tumors were harvested six hours following the final dose of treatment on day 8. Representative images (40X objective) from technical repeats on tumors from two mice per group are shown.
